# A study of the spatial correlation network structure of urban innovation in Guangdong

**DOI:** 10.1371/journal.pone.0272026

**Published:** 2022-09-23

**Authors:** Wei Liu, Qiuling Chen, Yue Wang

**Affiliations:** 1 School of Economics, Shanghai University, Shanghai, China; 2 Business School, University of Jinan, Jinan, China; Szechenyi Istvan University: Szechenyi Istvan Egyetem, HUNGARY

## Abstract

Based on the modified gravity model, a spatial correlation network of innovation was constructed among cities in Guangdong, China. Social network analysis was employed to explore their evolution characteristics during 2009–2017. The results indicate that the innovation output of prefecture-level cities in Guangdong Province shows both spatial correlations and differences. Their network shows lower density, higher efficiency, and rigid stratification properties. Based on small cluster analysis, these cities are classified into four blocks, the members of which changed. In 2017, four well-defined subgroups formed, which are “bidirectional spillover plate”, “main spillover plate”, “net beneficial plate”, and “agent plate”. With this network, the geographical characteristics of the innovation capabilities and differences among the cities in Guangdong, as well as the different positions and roles of each city in the associated network, can be properly understood. Consequently, the transmission mechanisms and development strategies of innovation in Guangdong Province can be better explored.

## Introduction

Guangdong Province always ranked first in China in terms of economic development. Its strategic industrial clusters generate agglomeration effects, while investing heavily in R&D expenditure. According to the 2020 Guangdong Provincial government Work Report, the proportion of regional GDP increased from 2.4% to 2.9%, and Guangdong also holds the first place in China in terms of overall regional innovation capacity. However, the development of Guangdong’s technological innovation system still faces the dilemma of having a weak foundation, insufficient accumulation of innovation resources, and uneven spatial distribution. There is enormous room for improvement of the future technological innovation development. In the current context of regional integration and development, cities form concentration areas of innovation drive and are the main carriers for implementing the innovation drive strategy. For a long time, regional differences have been apparent because of differences in the level of economic development, regional policies, and innovation resource endowment. In 2019, China promulgated the Outline of the Development Plan of the Guangdong-Hong Kong-Macao Greater Bay Area, which has led to an increasingly close exchange of innovation resources among cities in Guangdong Province. The diffusion of innovative achievements is increasingly accelerating, and the spatially linked network of technological innovation among cities has complex structural characteristics. The innovation network can effectively enhance the level of urban innovation through efficient resource allocation [1].

In this paper, the evolution of the spatial pattern and network structural characteristics of urban innovation in Guangdong Province is explored. The dynamics and evolution mechanism of urban innovation development are identified, and a synergistic spatial development plan is proposed. This plan can accelerate the overall strength of technological innovation in the cities of Guangdong in the future, which is of great practical value.

## Literature review

The new economic geography theory argues that spatial diffusion of innovation outputs can effectively alleviate innovation imbalance between regions and, in turn, narrow the innovation development gap between regions [2]. At present, many studies focus on innovation in improving the measurement methods, the status and evolution of linkage patterns, and relevant influencing factors [3–5].

Two main models are employed for measuring innovation linkages: one measures the strength of urban innovation linkages through improved gravity models [6], the other uses data such as patent cooperation to construct urban technology innovation networks [7–9]. The advantage of the former is that it is based on current and easily accessible panel data, reflecting the latest trends in the study population, and considering economic and geographical factors. It can therefore not only measure the relevance of the region as a whole but also the spatial transmission paths between individuals in the region. Essentially, possible innovation linkages can be modeled based on inter-city distances [10]. In contrast, using patent cooperation data can reflect technological linkages between cities in a relatively realistic way; the downside is that cooperation data cannot characterize the dynamics of factor flows.

There are two directions in research on spatial innovation patterns: Firstly, the current state of spatial regional innovation patterns is studied, using methods such as the Moran index [11–13], Granger causality tests [14], locational Gini coefficients, and statistical methods based on Lorentz curves and coefficients of variation [15, 16]. Breschi (2000) argued that significant European innovation technologies exist in the spatial innovation pattern and that there are significant similarities between countries in the spatial pattern of innovation in each technological stratum [17]. Spatial agglomeration of innovation output between provincial regions in China is strong, and exhibits strong spatially dependent characteristics at the global level [18]. The spatial pattern in China is characterized by unevenness and spatial heterogeneity [19–21]. These studies widely uncovered a spatial non-equilibrium under a high concentration of innovation activities, indicating the scale-free nature of the spatial distribution of innovation activities. There are fewer empirical studies on the level of urban innovation development and the characteristics of its pattern within a specific region, or on the evolution of innovation networks and their spatial structure. Secondly, spatial pattern evolution characteristics of innovation are studied. The spatial heterogeneity of innovation links and their evolution characteristics are explored from different scales using social network analysis (SNA) and exploratory spatial data analysis [10, 22, 23], showing that the “Matthew effect” is common in the network [24]. While ESDA can be used to disclose the spatial clustering and distribution of innovation among cities, it cannot identify the complex asymmetric linkage structure of innovation in each city on a larger scale. Moreover, it cannot clearly portray the role and function of each city in the overall spatial network and cannot precisely locate the center of gravity of innovation development. Based on patent cooperation data, Wang et al. (2019) used SNA and found that innovation linkages in the cities of the Yangtze River Delta gradually formed a “core-edge” network structure [25].

In terms of influencing factors, geographical proximity, innovation policies, foreign direct investment, firm size, industrial clusters, and innovation environment are the main factors affecting the level of regional innovation [26–29]. Based on 286 Chinese cities above prefecture level, Ziming (2018) showed that institutional factors, education level, external capital elements, industrial structure, and infrastructure all have significant positive effects on the status of city node networks [30].

The contributions of this paper are summarized in the following: The gravitational force model is modified from the perspective of spatial association, and SNA is used to assess the patent output of cities in Guangdong Province from 2009 to 2017. Patent output is an indicator of innovation development dynamics, and the overall network nature, node centrality, small group identification, and interactive relationship characteristics are measured according to three aspects. The temporal dimension is studied in depth. The dynamic evolution of inter-city innovation links and the spatial pattern and structure of innovation networks are portrayed from a network perspective, and heterogeneous suggestions for the optimization and synergistic development of technological innovation in cities are proposed based on the roles each city assumes.

## Research design

There are clear administrative boundaries between cities in Guangdong. Their activities are interdependent and the spatial correlation network of innovation in each city does not present a simple linear correlation but likely presents a multi-linear complex network. If the entire province is considered as one network, this network can either provide opportunities for or limit the actions of cities. SNA can overcome the limitations of traditional individual ‘attribute’ data analysis by using ‘relationship’ data to describe the vectorial association characteristics of spatial networks. Then, the impact of network correlation on cities can be analyzed, and the influence of ‘personal variables’ between actors on network-wide actions can be explored.

### Global spatial autocorrelation

Global spatial autocorrelation measures the degree of spatial correlation and spatial difference between regional units. The global Moran’s I index is commonly used as a calculation index for measuring global spatial autocorrelation. It reflects the degree of autocorrelation of the innovation elements of adjacent regional units in the global environment. Moran’s I is expressed as follows:

$$Moran^{\prime }s\;I=\frac{\sum_{i=1}^{n}\sum_{j=1}^{n}w_{ij}\left(x_{i}-\bar{x}\right)\left(x_{j}-\bar{x}\right)}{s^{2}\sum_{i=1}^{n}\sum_{j=1}^{n}w_{ij}}$$ (1)

where *I* is the global spatial autocorrelation index, *s* is the standard deviation, *w*_*ij*_ is the spatial connection matrix of spatial units *i* and *j* in the study range, *n* is the total number of samples, *x*_*i*_ and *x*_*j*_ are sample values, and $\bar{x}$ is the average value. The spatial connection matrix is constructed according to the adjacency of regional units, i.e., if there is a common boundary between regions *i* and *j*, belonging to the neighbor relationship, *w*_*ij*_ = 1; otherwise *w*_*ij*_ = 0. The range of Moran’s I is [-1,1]. If *I* < 0, the correlation between urban innovation is negative; if *I* = 0, the urban innovation is not related; if *I* > 0, the correlation between urban innovation is positive.

*I* can be standardized, and *Z* is used to represent its normalized statistics. The formula is:

$$Z=\frac{I-E\left(I\right)}{\sqrt{VAR\left(I\right)}}$$ (2)

The meaning of the *Z* value is the same as the global Moran’s I index.

### Construction method of spatial association network

With the continuous development of Economics, Liu (2019) [31], Cao (2019) [32], and other scholars introduced the gravity model to the study of economics. Therefore, in this paper, an improved gravity model is adopted to construct a spatial correlation network of regional innovation output.

The level of economic development and the number of R&D personnel are introduced as the influencing factors of urban innovation output. Because of the good economic development momentum of this city, the urban innovation environment is well developed, the number of R&D personnel is large, and their quality is high. This leads to high technical strength and enhanced urban innovation. Through the above analysis, the factors affecting the urban innovation of Guangdong are included in the improved gravity model. The formula is:

$$G_{ij}=\frac{P_{i}}{P_{i}+P_{j}}\frac{\sqrt[{4}]{P_{i}H_{i}I_{i}E_{i}}\sqrt[{4}]{P_{j}H_{j}I_{j}E_{j}}}{D_{ij}{}^{2}}$$ (3)

where, the meaning of each symbol is detailed in Table 1.

**Table 1:** Meaning of symbols in Eq (3).

Symbol	Meaning description
*i*, *j*	cities in Guangdong
*G*	Gravity between prefecture-level cities in Guangdong
*P*	Total output of innovation
*H*	Number of R&D staff
*I*	R&D investment
*E*	GDP per capita
*D*	Distance between prefecture-level cities in Guangdong

The steps for constructing the spatial association network are as follows. First, the gravity matrix is calculated according to Eq (3). Second, the average of each row of the gravity matrix is averaged and assigned a value of *g*. In the third step, the sizes of *G*_*ij*_ and g are compared. If *G*_*ij*_ is greater than g, a value of 1 is assigned, indicating that there is an association relationship; otherwise, a value of 0 is assigned, indicating that there is no association relationship. In the fourth step, after comparing the sizes, a 0–1 matrix is obtained, which is a spatial correlation matrix. A line with an arrow is drawn between the s with the value of 1, and a spatially associated network map is obtained.

### Characterization of the associated network

SNA is used to construct a spatial correlation network diagram of urban innovation based on the data processing of the gravity model. This diagram can quantitatively depict the correlation characteristics of urban innovation through network density, connectedness, and centrality analysis. The block models are used to qualitatively analyze the positions and roles of different cities in the spatial correlation network of innovation. The specific indicators are as follows:

1. Network density
  Network density reflects the density of the entire spatial association network. The value range of this measure is [0,1], and it is calculated via Eq (4):
$$D=\frac{R}{T}$$ (4)
  where, *D* represents the network density, *R* represents the actual number of network associations in the network, and *T* represents the number of network associations that should theoretically exist in the network.
2. Connectedness analysis
  If there are connections between any two cities in a network, the network is spatially connected. Measured by the connectedness index, the value range of this measure is [0,1], and the larger the value, the higher the correlation. The formula is as follows:
$$D_{c}=1-\frac{A}{T/2}$$ (5)
  where, *D*_*c*_ represents the degree of spatial association, *A* represents the number of unconnected points in the network, and *T* represents the number of network associations that should exist in the network theoretically, i.e., the theoretically reachable network that should exist in the network point logarithm.
3. Centrality analysis
  Centrality analysis is used to study the status and role of a certain area in the network. Generally, the function of the centrally located area should be the most important in the entire network. The more it can realize diffusion between areas, the more it can drive the development of the entire area.
  Point centrality is the number of other points that are directly connected to a specific point of interest. The higher the point centrality of this point, the more central it is in the network and the easier it is for it to obtain resources. In a directed network, the degree of each point can be divided into in-centrality and out-centrality:
$$C_{AD}(i)=\frac{C_{1}+C_{2}}{2(N-1)}$$ (6)
  where, *C*_*AD*_ represents the point centrality, *C*_1_ represents in-centrality, and *C*_2_ represents out-centrality.

The sociologist Lyndon Freeman (1978) introduced the concept of centrality [33]. If an actor is located between multiple pairs of actors, it may play an important ‘intermediary’ role and therefore be at the center of the network. Betweenness centrality is used to measure the ability of cities in the network to control resources:

$$C_{B}(i)=\sum_{j}^{n}\sum_{k}^{n}\frac{g_{jk}(i)}{g_{jk}},j\neq k\neq i,j<k$$ (7)

where, *g*_*jk*_ represents the shortest number of paths between point *j* and point *k*, and *g*_*jk*_ (*i*) represents the number of shortcuts between points *j* and *k* that lead through point *i*.

Closeness centrality indicates that the closer a point is to other points, the less dependent it is on others:

$$C_{c}=\frac{\sum_{j=1}^{N}d_{ij}}{M-1}$$ (8)

where, *C*_*c*_ represents closeness centrality, *d*_*ij*_ is the shortcut distance between nodes *i* and *j* (the number of lines included in the shortcut), M-1 represents the smallest proximity centrality in the network, and *M* is the area number.

### Block models

Block models are introduced into the structural division of the spatial correlation network of urban innovation. The relationship of the individual members is analyzed from the location *G*_*k*_. Assuming that there are *g*_*k*_ actors, the total number of possible relationships within *G*_*k*_ is *g*_*k*_(*g*_*k*_−1). There are g actors in total, and all possible relationships for each member of the *G*_*k*_ position are *g*_*k*_(*g*−1). The expected proportion of the total relationship for a position is: *g*_*k*_(*g*_*k*_−1)/*g*_*k*_(*g*−1) = (*g*_*k*_−1)/(*g*−1).

Based on the relationships within and between locations, this indicator can be divided into four types of spatial correlation network structure of urban innovation, as shown in Table 2.

**Table 2:** Four position types in block models.

Relationship ratio within the location	Proportion of relationship received by location	
	≈0	>0
≥ (*g*_*k*_−1)/(*g*−1)	bidirectional spillover plate	net beneficial plate/ main beneficial plate
< (*g*_*k*_−1)/(*g*−1)	net spillover plate	agent plate

In this paper, the block model analysis method is used to study the spatial correlation characteristics of Guangdong’s urban innovation. Furthermore, the degree of correlation of the spatial correlation network of innovation is tested, which helps to further analyze the geographic characteristics of urban innovation.

### Data sources

How to measure innovation has been controversially discussed in academia [34–38]. Indicators such as patents [15, 16, 39, 40], academic publications, and new product output can all reflect innovation. Because patent data has the characteristics of strong comparability, large amount of information, and easy accessibility, it has become the most widely used index. Therefore, the number of invention patent applications was selected as an indicator of urban innovation.

The data on the number of invention patent applications originate from the 2009–2018 Guangdong Statistical Yearbook on Science and Technology, Guangdong Intellectual Property Yearbook and the statistical bulletins of various prefecture-level cities in Guangdong. The data on economic development level of prefecture-level cities in Guangdong per capita GDP, the number of R&D personnel, and R&D investment funds originate from the Guangdong Statistical Yearbook. When using Eq (3) to construct the spatial correlation matrix of regional innovation output from 2008 to 2017, to avoid excessive data fluctuations and eliminate heteroscedasticity, economic data is uniformly logarithmically processed.

## Empirical analysis

The data on invention patent applications from 2008 to 2017 in 21 cities of Guangdong were imported into the ArcGIS spatial database, and global Moran’s I spatial autocorrelation analysis was carried out. The results are shown in Table 3.

**Table 3:** Global spatial correlation test results.

Year	*Moran’s I*	Z	P
2008	0.039275	1.684678	0.092051
2009	0.056175	1.795023	0.07265
2010	0.098682	2.239462	0.025126
2011	0.11425	2.359799	0.018285
2012	0.151635	2.597622	0.009387
2013	0.193206	2.730164	0.00633
2014	0.259519	2.937144	0.003313
2015	0.307723	3.152821	0.001617
2016	0.336176	3.202235	0.001364
2017	0.359592	3.191962	0.001413

As shown in Table 3, the Moran’s I indices from 2008 to 2017 all pass the 10% significance test, indicating that there is a positive spatial correlation between urban innovation in each city in Guangdong, with spatial clustering characteristics. Overall, the value of Moran’s I in Guangdong increased from 0.04559 in 2008 to 0.358856 in 2017, indicating that the urban accumulation of innovation has followed an increasing trend and is becoming more significant. During this period, innovative links between cities with low innovation capabilities and cities with high innovation capabilities have gradually increased, and gathering in space has also increased.

## Spatial correlation

### Spatial correlation network

According to Eq (3), as shown in Table 4, a 0–1 matrix of innovation for 21 cities in Guangdong in 2017 can be derived.

**Table 4:** Modal network of 21 cities in Guangdong.

**Number**	**City**	**1**	**2**	**3**	**4**	**5**	**6**	**7**	**8**	**9**	**10**	**11**	**12**	**13**	**14**	**15**	**16**	**17**	**18**	**19**	**20**	**21**
1	Guangzhou	0	1	0	0	1	0	0	0	1	0	1	1	0	0	0	0	0	0	0	0	0
2	Shenzhen	1	0	1	0	1	0	0	0	1	0	1	1	0	0	0	0	0	0	0	0	0
3	Zhuhai	0	1	0	0	1	0	0	0	0	0	1	1	1	0	0	0	0	0	0	0	0
4	Shantou	0	0	0	0	0	0	0	1	0	1	0	0	0	0	0	0	0	0	1	1	0
5	Foshan	1	1	1	0	0	0	0	1	0	0	1	1	1	0	0	0	0	0	0	0	0
6	Shaoguan	1	0	0	0	1	0	1	0	1	0	1	1	0	0	0	0	1	1	0	0	0
7	Heyuan	0	0	0	1	0	1	0	0	1	1	0	0	0	0	0	0	0	0	1	1	0
8	Meizhou	0	0	0	1	0	0	1	0	0	1	0	0	0	0	0	0	0	0	1	1	0
9	Huizhou	1	1	0	0	1	0	0	0	0	0	1	1	0	0	0	0	0	0	0	0	0
10	Shanwei	0	0	0	1	0	0	1	0	1	0	0	0	0	0	0	0	0	0	1	1	0
11	Dongguan	1	1	0	0	1	0	0	0	1	0	0	1	0	0	0	0	0	0	0	0	0
12	Zhongshan	1	1	1	0	1	0	0	0	0	0	1	0	1	0	0	1	0	0	0	0	0
13	Jiangmen	1	1	1	0	1	0	0	0	0	0	1	1	0	0	0	1	0	0	0	0	0
14	Yangjiang	0	0	0	0	0	0	0	0	0	0	0	1	1	0	0	1	1	0	0	0	1
15	Zhangjiang	0	0	0	0	0	0	0	0	0	0	0	1	1	1	0	0	1	0	0	0	1
16	Maoming	0	0	1	0	1	0	0	0	0	0	0	1	1	1	1	0	1	0	0	0	1
17	Zhaoqing	1	0	1	0	1	0	0	0	0	0	1	1	1	0	0	0	0	1	0	0	1
18	Qingyuan	1	0	0	0	1	1	0	0	1	0	1	1	0	0	0	0	1	0	0	0	0
19	Chaozhou	0	0	0	1	0	0	0	1	0	0	0	0	0	0	0	0	0	0	0	1	0
20	Jieyang	0	0	0	1	0	0	0	1	0	1	0	0	0	0	0	0	0	0	1	0	0
21	Yunfu	0	0	0	0	1	0	0	0	0	0	0	0	1	1	0	1	1	0	0	0	0

Note: The numbers in the first row are expressed as prefecture-level cities in Guangdong

**Fig 1:**
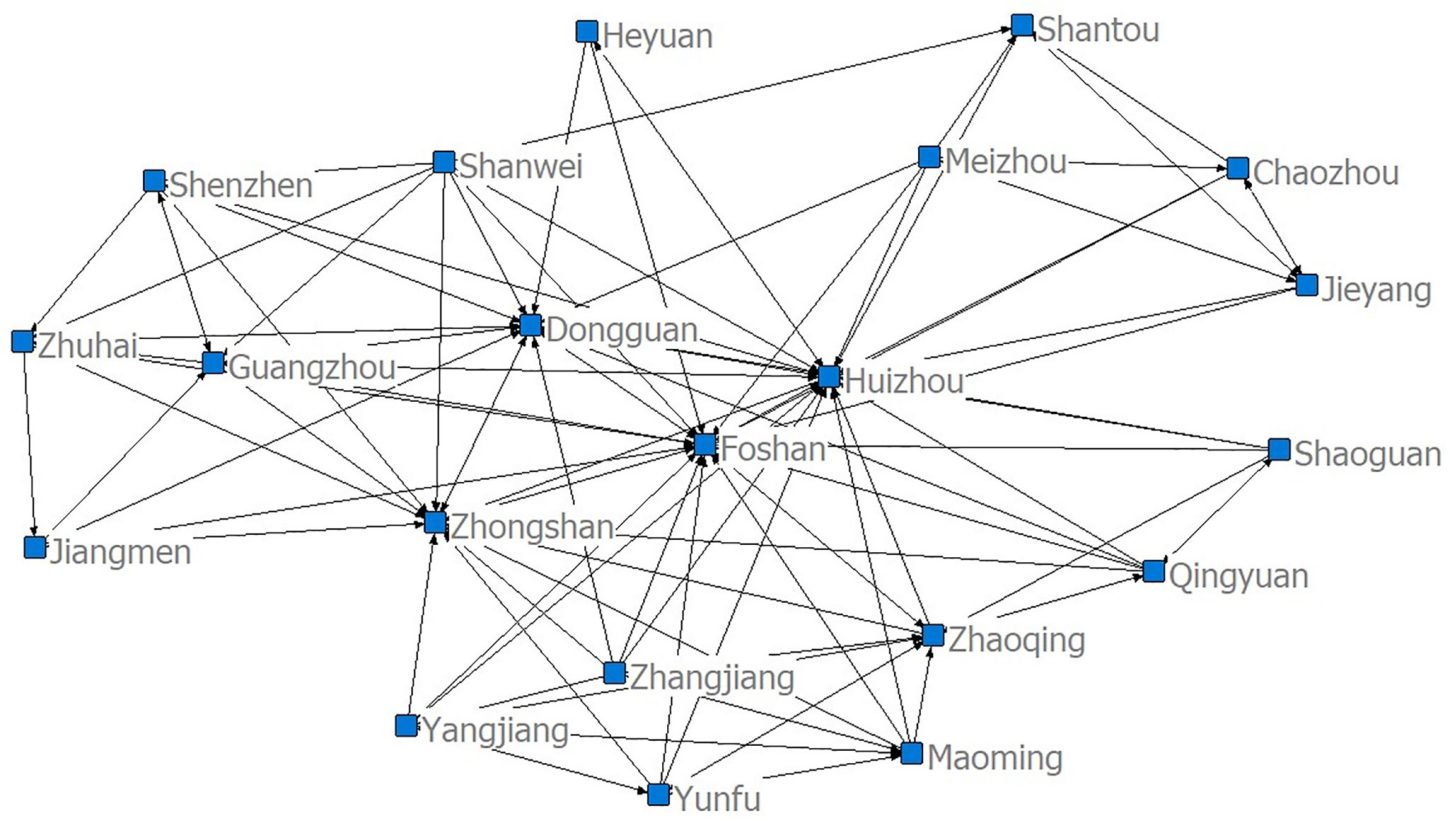
Spatial correlation network of urban innovation in Guangdong.

According to the data in Table 4, innovation in cities of Guangdong is spatially correlated, as shown in Fig 1.

The spatial correlation of Guangdong is complex, and there is a general correlation between innovation in each city. Dense and large areas of the network in the graph indicate that this city is at the core position in the spatial correlation network of innovation. This is a “symbol of power” of this city, indicating that its participation in regional innovation output activities is the most active. Cities with high activity are those with developed economies in Guangzhou, Shenzhen, and Dongguan. However, the cities of Yangjiang and Zhongshan have mainly suffered from spatial spillover of innovation in other cities.

The urban spatial correlation relationship mainly includes spatial spillover and spatial benefit. A comparative analysis of the urban innovation correlation in Guangdong is shown in Table 5.

**Table 5:** Statistical results of the correlation between urban innovation.

City	Beneficial relationship	Overflow relationship	Total number of associations
Guangzhou	5	9	14
Shenzhen	6	7	13
Zhuhai	5	6	11
Shantou	4	5	9
Foshan	7	12	19
Shaoguan	8	2	10
Heyuan	6	3	9
Meizhou	5	4	9
Huizhou	5	7	12
Shanwei	5	4	9
Dongguan	5	10	15
Zhongshan	7	13	20
Jiangmen	7	8	15
Yangjiang	5	3	8
Zhangjiang	5	1	6
Maoming	8	4	12
Zhaoqing	8	6	14
Qingyuan	7	2	9
Chaozhou	3	5	8
Jieyang	4	5	9
Yunfu	5	4	9

In the spatial spillover of urban innovation, in 2017, top-ranked cities were Zhongshan, Foshan, Dongguan, Guangzhou, Jiangmen, Shenzhen, and Huizhou. Among them, Zhongshan, Foshan, Dongguan, and Guangzhou have more than nine spillover relationships. Guangzhou is the capital of Guangdong, which has excellent, geographical location, political, economic, scientific, and technological culture, as well as universities and research institutions, and a strong diffusion effect. Because of good economic conditions, investment in science and technology, convenient transportation, and preferential policies, Zhongshan exerts a spillover effect on innovation comparable to that of Guangzhou. The role of Shenzhen, which has advantages in all aspects of economic development, has a relatively weak innovation diffusion. Meizhou, Heyuan, Shanwei, Zhanjiang, Zhaoqing, Qingyuan, Yangjiang, and Maoming have weak innovation and innovation diffusion effect. These cities have low levels of economic development, transportation, and informatization.

### Network density

**Fig 2:**
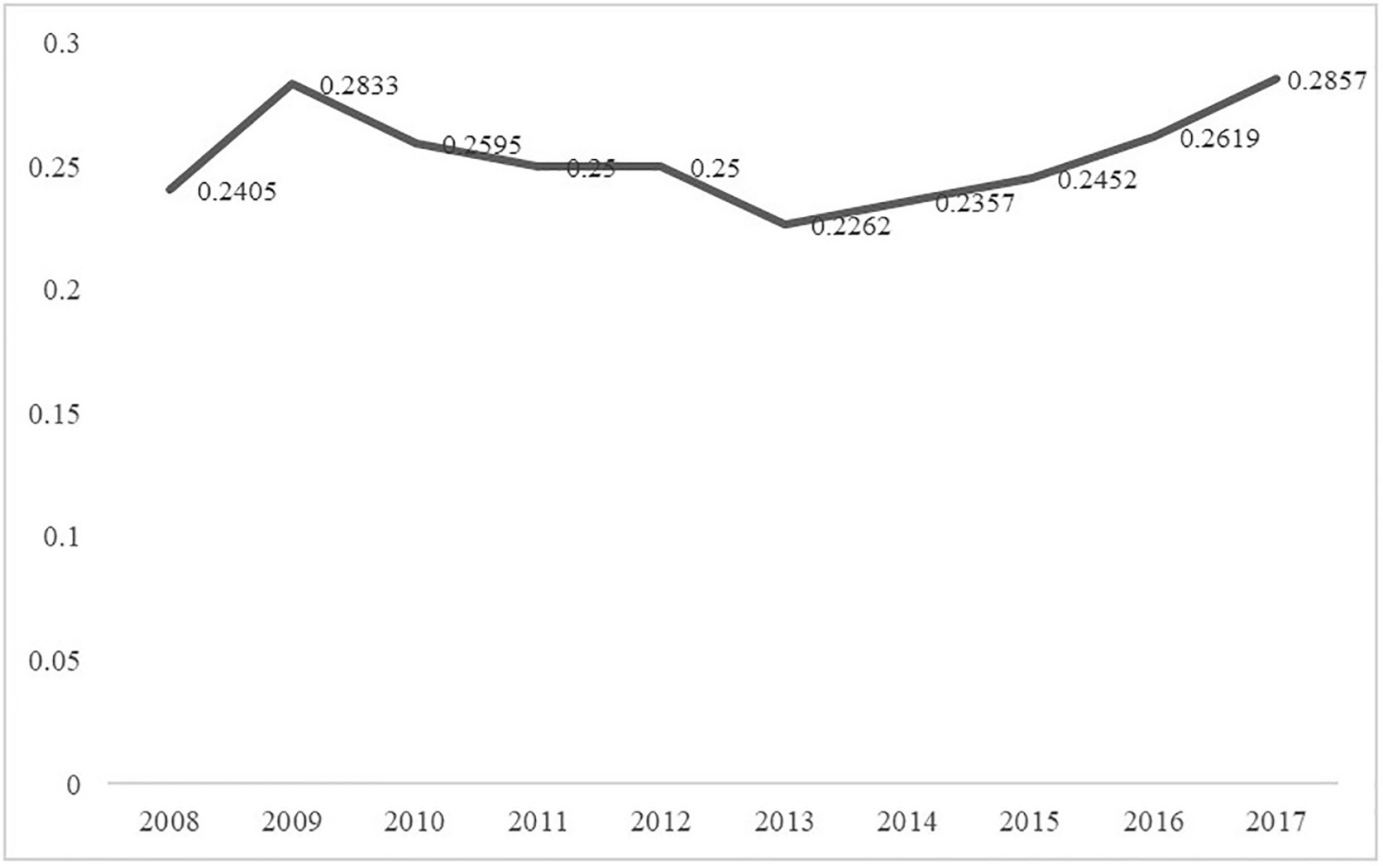
Network density of urban innovation in Guangdong.

Based on Eq (4), the overall network density from 2008 to 2017 were calculated as shown in Fig 2.

The network density and connectedness both show an increasing trend. The urban innovation network density in Guangdong has increased from 0.2405 in 2008 to 0.2857 in 2017, indicating that the spatial correlation network of urban innovation in Guangdong is dynamically improving. Cities are increasingly connecting with each other. However, low-density values and the slow growth rate of the network are far from saturated in terms of closeness, which manifests the spatial correlation of regional innovation in Guangdong.

### Connectedness analysis

**Fig 3:**
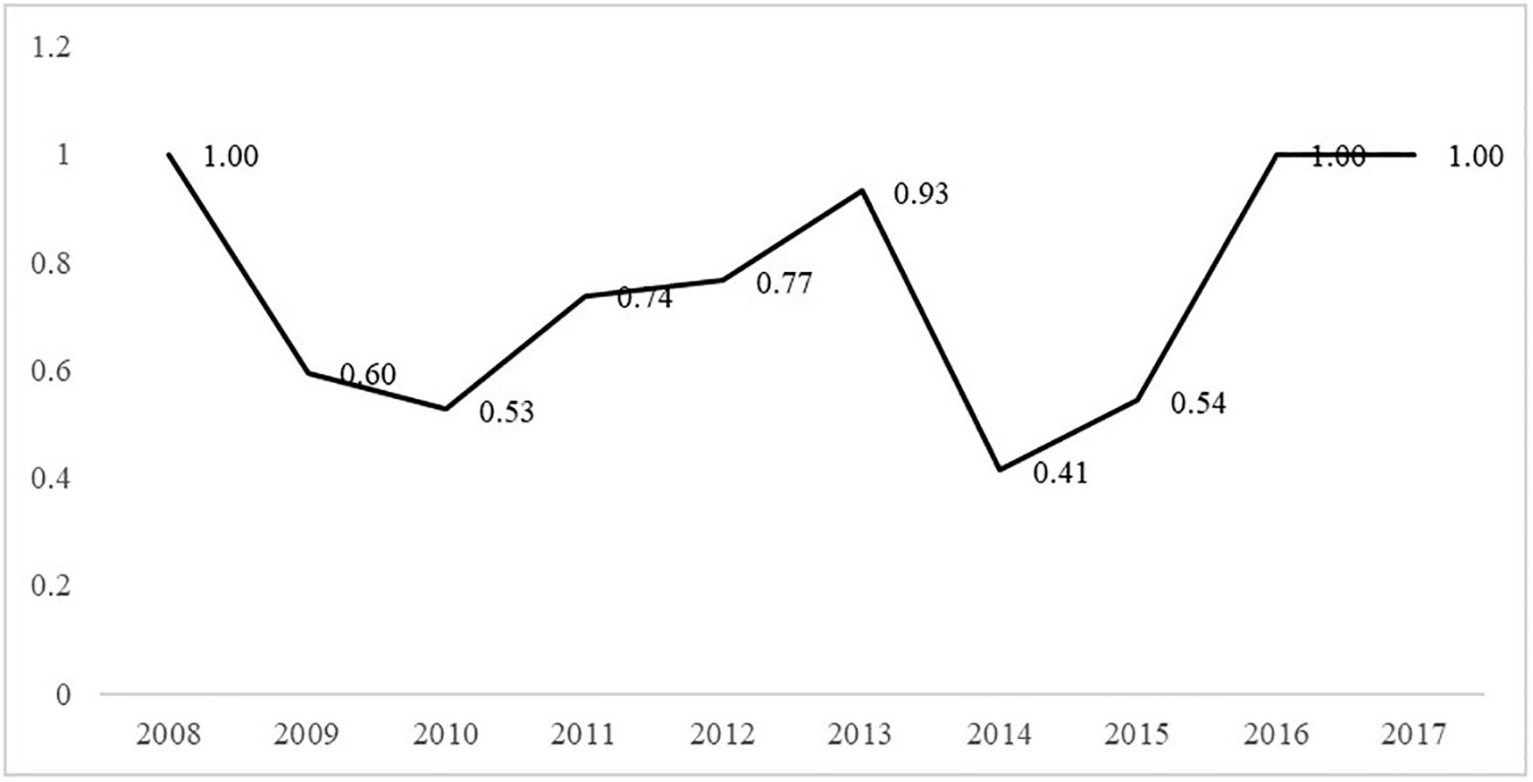
Connectedness of urban innovation in Guangdong.

Based on Eq (5), the overall network connectedness from 2008 to 2017 were calculated as shown in Fig 3.

The degree of correlation fluctuates from high to low, showing a “W” shape. The correlation degree changed from 1 in 2008 to 0.5285 in 2010. The spillover effect of regional innovation in Guangdong decreased during these three years. By 2013, the connectedness had again increased to 1. The connectedness in 2014 decreased, and the correlation slowly increased to 1 starting in 2017. This shows that there is a general spatial spillover relationship between urban innovation in Guangdong, but the spillover effect is unstable.

According to Eq (5), the paper use UCINET software to construct the reachability matrix of the spatial correlation network, further analysing connectedness of the spatial correlation network of Guangdong’s urban innovation in 2017. As shown in Table 6:

**Table 6:** Reachability matrix of the spatial correlation network.

	City	1	2	3	4	5	6	7	8	9	10	11	12	13	14	15	16	17	18	19	20	21
1	Guangzhou	0	1	1	1	1	1	1	1	1	1	1	1	1	1	1	1	1	1	1	1	1
2	Shenzhen	1	0	1	1	1	1	1	1	1	1	1	1	1	1	1	1	1	1	1	1	1
3	Zhuhai	1	1	0	1	1	1	1	1	1	1	1	1	1	1	1	1	1	1	1	1	1
4	Shantou	1	1	1	0	1	1	1	1	1	1	1	1	1	1	1	1	1	1	1	1	1
5	Foshan	1	1	1	1	0	1	1	1	1	1	1	1	1	1	1	1	1	1	1	1	1
6	Shaoguan	1	1	1	1	1	0	1	1	1	1	1	1	1	1	1	1	1	1	1	1	1
7	Heyuan	1	1	1	1	1	1	0	1	1	1	1	1	1	1	1	1	1	1	1	1	1
8	Meizhou	1	1	1	1	1	1	1	0	1	1	1	1	1	1	1	1	1	1	1	1	1
9	Huizhou	1	1	1	1	1	1	1	1	0	1	1	1	1	1	1	1	1	1	1	1	1
10	Shanwei	1	1	1	1	1	1	1	1	1	0	1	1	1	1	1	1	1	1	1	1	1
11	Dongguan	1	1	1	1	1	1	1	1	1	1	0	1	1	1	1	1	1	1	1	1	1
12	Zhongshan	1	1	1	1	1	1	1	1	1	1	1	0	1	1	1	1	1	1	1	1	1
13	Jiangmen	1	1	1	1	1	1	1	1	1	1	1	1	0	1	1	1	1	1	1	1	1
14	Yangjiang	1	1	1	1	1	1	1	1	1	1	1	1	1	0	1	1	1	1	1	1	1
15	Zhanjiang	1	1	1	1	1	1	1	1	1	1	1	1	1	1	0	1	1	1	1	1	1
16	Maoming	1	1	1	1	1	1	1	1	1	1	1	1	1	1	1	0	1	1	1	1	1
17	Zhaoqing	1	1	1	1	1	1	1	1	1	1	1	1	1	1	1	1	0	1	1	1	1
18	Qingyuan	1	1	1	1	1	1	1	1	1	1	1	1	1	1	1	1	1	0	1	1	1
19	Chaozhou	1	1	1	1	1	1	1	1	1	1	1	1	1	1	1	1	1	1	0	1	1
20	Jieyang	1	1	1	1	1	1	1	1	1	1	1	1	1	1	1	1	1	1	1	0	1
21	Yunfu	1	1	1	1	1	1	1	1	1	1	1	1	1	1	1	1	1	1	1	1	0

Note: 1–21 in the second row represents the prefecture-level city in the first column

It can be concluded that the unreachable area pair in the network is 0. According to Eq (5), the connectedness of the spatial correlation network of urban innovation in Guangdong is 1, which is quite high. This indicates that it has a relatively good effect. In this network, the spatial spillover effect between prefecture-level cities is universal, and the accessibility between cities in the overall network is good.

The network density is 0.2857 in 2017, but the connectedness is strong. The correlation between all possible cities and cities is also strong, but the overflow level between each city is relatively low. The overall network structure is relatively loose. There is little real “communication” between the innovation of various cities in Guangdong, and the quality of cooperation between cities should be strengthened.

### Centrality analysis

To analyze the spatial relevance of urban innovation in Guangdong, UCINET software was used and the point centrality, betweenness centrality, and closeness centrality were calculated for each prefecture-level city in Guangdong.

#### Analysis of point centrality

A city that has more direct relationships with other cities is at the center of the network, and has a higher point centrality and greater power. This city has a significant innovation spillover and agglomeration effect. Point centrality is mainly divided into the two categories of absolute and relative point centrality. The latter is a standardized form of the former. UCINET software was used to calculate the relative degree centrality of 21 prefecture-level cities in Guangdong, and the results are shown in Table 7:

**Table 7:** Point centrality of the spatial correlation network.

City	OutDegree	InDegree	Sum	NrmOutDeg	NrmInDeg
Guangzhou	5	9	14	25	45
Zhongshan	7	13	20	35	65
Huizhou	5	7	12	25	35
Zhuhai	5	6	11	25	20
Foshan	7	12	20	35	60
Dongwan	5	10	15	25	50
Shenzhen	6	7	13	30	35
Meizhou	5	4	9	25	20
Jiangmen	7	8	15	35	40
Shanwei	5	4	9	25	20
Shaoguan	8	2	10	40	10
Yunfu	5	4	9	25	20
Qingyuan	7	2	9	35	10
Shantou	4	5	9	20	25
Zhanjiang	5	1	6	25	5
Heyuan	6	3	9	30	15
Zhaoqing	8	6	14	40	30
Yangjiang	5	3	8	25	15
Maoming	8	4	12	40	20
Jieyang	4	5	9	20	25
Chaozhou	3	5	8	15	25
Max	4	11	14	20	55
Min	0	0	1	0	0
Average	5.714	5.714	11.476	28.571	28.571
Standard deviation	1.385	3.149	3.776	6.925	15.747
Network centrality	12.000%	38.250%			

The standard deviation of the spillover relationship in the spatial correlation network of the urban innovation output in Guangdong is 1.385, and the standard deviation of the benefit relationship is 3.149. This shows that there is a large difference among the spatial benefit relationship of urban innovation in Guangdong. It also shows that the beneficiary relationships in certain regions are relatively concentrated. The main cities with a high concentration of these beneficiary relationships are Shaoguan, Maoming, and Zhaoqing. Specifically, Zhongshan, Foshan, and Dongguan have many spillover relationships, all of which exceeding 10, while Zhanjiang has the lowest spillover relationship of 1. Zhongshan, Foshan, Dongguan, and Guangzhou are located at the center of the regional innovation network. These cities rely on their beneficial geographic location or developed economy, which have diffusion and agglomeration functions. These not only spill over to the outside, but these cities also continuously absorb knowledge and enhance their own innovation capabilities. The centrality of Guangdong’s urban innovation spillover is 38.25%, while the centrality of the beneficiaries is 12%, indicating that asymmetry exists between the spillover and the benefit of Guangdong’s urban innovation. The central power of the star network is 100%, indicating that the closer the central power is to 1, the more centralized the network is. From the point of InDegree in Table 7, the entire spatial network centrality of urban innovation in Guangdong has a slightly larger centrality, and the spatial network has a certain tendency to concentrate.

#### Betweenness centrality

The betweenness centrality of 21 cities in Guangdong in 2017 is calculated according to Eq (7). The specific values are shown in Table 8.

**Table 8:** Betweenness centrality of the spatial correlation network.

City	Betweenness	nBetweenness
Foshan	93.714	24.662
Meeizhou	89.970	23.676
Zhongshan	71.447	18.802
Maoming	61.150	16.092
Huizhou	51.532	13.561
Heyuan	13.000	11.461
Shanwei	37.948	9.986
Zhaoqing	35.492	9.340
Shaoguan	31.068	8.176
Jiangmen	16.631	4.377
Qingyuan	7.605	2.001
Yunfu	6.733	1.772
Shenzhen	5.308	1.397
Dongguan	3.957	1.041
Shantou	3.248	0.855
Jieyang	3.248	0.855
Guangzhou	3.186	0.838
Zhuhai	1.093	0.288
Chaozhou	0.667	0.175
Yangjiang	0.450	0.118
Zhanjiang	0.000	0.000
Max	93.714	24.662
Min	0.000	0.000
Average	27.048	7.118
Standard deviation	30.104	7.922
Network centrality		18.42%

The standard deviation of the centrality of the spatial correlation network of urban innovation in Guangdong is 7.922, which is relatively large, indicating that the spatial correlation network of urban innovation in Guangdong is considerably imbalanced. The standardization intermediate trend of the entire network is 18.42%, indicating low ‘intermediate centrality’ of the network. Specifically, Foshan, Meizhou, Zhongshan, Maoming, Huizhou, and Heyuan all have a relative betweenness centrality of over 10%. The betweenness centrality levels of Foshan, Meizhou, Zhongshan, Maoming, Huizhou, Shanwei, Zhaoqing, and Shaoguan all exceed 30. This indicates that Foshan, Meizhou, Zhongshan, Maoming, Huizhou and Heyuan have a certain “bridging” role in the spatial correlation network of urban innovation in Guangdong. These cities have a strong ability to disperse and agglomerate other cities, and therefore play a major role in the spatial correlation network of urban innovation. As shown in Fig 1, Foshan, Meizhou, Zhongshan, Maoming, and Huizhou are at the center of the network.

#### Closeness centrality

The closeness centrality of the spatial correlation network of urban innovation in Guangdong was calculated according to Eq (8), and the results are shown in Table 9.

**Table 9:** Closeness centrality of the spatial correlation network.

City	inFarness	outFarness	inCloseness	outCloseness
Zhongshan	32.000	41.000	62.500	48.780
Foshan	33.000	40.000	60.606	50.000
Dongguan	35.000	48.000	57.143	41.667
Guangzhou	36.000	48.000	55.556	41.667
Meizhou	38.000	56.000	52.632	35.714
Huizhou	38.000	48.000	52.632	41.667
Shenzhen	38.000	47.000	52.632	42.553
Jiangmen	43.000	41.000	46.512	48.780
Zhuhai	45.000	48.000	44.444	41.667
Maoming	47.000	39.000	42.553	51.282
Zhaoqing	48.000	38.000	41.667	52.632
Jieyang	50.000	61.000	40.000	32.787
Shantou	50.000	61.000	40.000	32.787
Chaozhou	50.000	73.000	40.000	27.397
Shanwei	51.000	46.000	39.216	43.478
Heyuan	51.000	42.000	39.216	47.619
Yunfu	56.000	42.000	35.714	47.619
Qingyuan	60.000	39.000	33.333	51.282
Shaoguan	60.000	34.000	33.333	58.824
Yangjiang	62.000	48.000	32.258	41.667
Zhanjiang	65.000	48.000	30.769	41.667
Max	65.000	73.000	48.000	58.824
Min	32.000	34.000	30.769	27.397
Average	47.048	47.048	44.415	43.883
Standard deviation	9.703	9.010	9.414	7.364
Intrenal closeness centralization	38.98%	External closeness centralization	32.20%	

The standard deviations of internal and external closeness centrality of the urban innovation spatial correlation network in Guangdong are 9.414 and 7.364, respectively, which is higher than that of external closeness centrality. In the urban innovation spatial association network of Guangdong in 2017, the imbalance of the beneficiary effect is greater than that of the spillover effect. The internal closeness centrality of Guangdong’s urban innovation is 38.98%, and the external closeness centrality is 32.20%. The closeness centrality has a relatively low level, indicating that in urban innovation delivery, the information transmitted by each city cannot be received by other cities in a timely and effective manner. The innovative collaboration capabilities of these 21 cities should be improved.

Furthermore, its relatively high ranking of “closeness centrality” indicates that this region is relatively independent. This indicates that it is relatively easy for the cities of Guangdong to receive and absorb innovative information from other cities. According to their closeness centrality, the top-ranking cities are Zhongshan, Foshan, Dongguan, Guangzhou, Meizhou, Huizhou, and Shenzhen, which have low external closeness centrality. This implies that these cities are more likely to accept the spatial spillover relationship of innovation. The resulting spatial spillover relationship is low, and the independence of acceptance is strong and not easily affected by other cities. The top cities in terms of their out closeness centrality are Zhongshan, Foshan, Jiangmen, Maoming, Zhaoqing, Qingyuan, and Shaoguan, which are more likely to have spatial spillover relationships and less spatial beneficiary relationships. This indicates the high validity of the spatial overflow relationship.

#### Block model analysis

In this paper, the spatial correlation between the urban innovation of Guangdong is further analyzed using block models.

According to block model, a maximum segmentation depth of 2 and a convergence criterion of 0.2 were chosen to divide the cities of Guangdong into four major segments using the CONCOR algorithm in Ucinet software (Table 10).

**Table 10:** Comparison of centrality analysis.

Centrality index	Secondary indicators	City
Point Centrality	InDegree	Shaoguan, Maoming, Zhaoqing
	OutDegree	Zhongshan, Fosha, Dongguan
Betweenness Centrality		Foshan, Meizhou, Zhongshan, Maoming, Huizhou, Shanwei, Zhaoqing, Shaoguan
Closeness Centrality	inCloseness	Zhongshan, Foshan, Dongguan, Guangzhou, Meizhou, Huizhou, Shenzhen
	outCloseness	Zhongshan, Foshan, Jiangmen, Maoming, Zhaoqing, Qingyuan, Shaoguan

According to Table 11, the cities included in the different blocks were counted, as was the number of relations received and sent. The data are shown in Table 12.

**Table 11:** Division of blocks.

Innovation Block	Number of Cities	Receiving Relationship		Sending Relationship		Ratio		Block Type
		Internal Block	External Block	Internal Block	External Block	EIRR (%)	ATRR (%)	
Block I	10	56	6	56	20	45	90	net spillover
Block II	5	14	17	14	4	20	45	main beneficial plate
Block III	4	12	4	12	7	15	75	"bidirectional spillover plate
Block IV	2	2	9	2	5	10	18	agent plate

Notes (expected internal relationship ratio (EIRR), actual internal relationship ratio (AIRR))

**Table 12:** Cities with spatial correlation.

Innovation Block	Cities	Receiving Relationship	Sending Relationship
Block I	Guangzhou, Shenzhen, Zhuhai, Dongwuan, Foshan, Jiangmeng, Zhongshan, Huizhou, Shaoguan, Qingyuan	62	76
Block II	Zhanjiang, Maoming, Yangjiang, Yunfu, Zhaoqing	31	18
Block III	Shantou, Jieyang, Chaozhou, Meizhou	16	19
Block IV	Shanwei, Heyuan	11	7

Combination of the data in Tables 11 and 12 shows that in Block I, there are 76 relationships, 20 of which belong to the relationship of internal block, and 6 relationships originate from other blocks. The expected internal relationship ratio is 45%, but the actual internal relationship ratio is 90%. Therefore, Block I belongs to the “net spillover plate”. Moreover, all members included in Block I belong to the developed regions of the Pearl River Delta. This also indicates that developed cities have strong innovation vitality and can generate a wide range of innovative spatial spillover relationships.

Block II generated 18 relationships, 14 of which were intra-board relationships and 17 were received from other boards. The expected internal relationship ratio was 20%, while the actual internal relationship ratio was 45%. Therefore, Block II was the “main beneficial plate”. The members of Block II were mainly cities in western Guangdong, which accept spillovers from the Pearl River Delta region.

Block III generated 19 relationships, 12 of which were intra-board relationships and 4 were received from other boards. The expected internal relationship ratio was 15%, while the actual internal relationship ratio was 75%. Therefore, Plate III was a typical “bidirectional spillover plate”. Block IV generated 7 relationships, 2 of which were intra-board relationships and 9 were received from other boards. The expected internal relationship ratio was 10%, while the actual internal relationship ratio was 18%. Therefore, Block IV was a typical “agent plate”.

According to the distribution of the correlations shown in Table 13, the density matrix of blocks can also be calculated to reflect the distribution of spillover effects in each innovation block.

**Table 13:** Density matrix of blocks.

	Block I	Block II	Block III	Block IV
Block I	0.622	0.340	0.000	0.150
Block II	0.080	0.700	0.000	0.000
Block III	0.025	0.000	1.000	0.750
Block IV	0.050	0.000	0.500	1.000

The spillover effect of Block I was mainly reflected in the internal Blocks I, II, and IV; the spillover effect of Block II was mainly reflected in the second internal Block; the spillover effect of Block III was primarily reflected in Blocks II and IV; the spillover effect of Block IV was mainly reflected in Block IV, and it also had a certain spillover effect on other blocks.

It shown in Table 14, the diagonals of the image matrix were all 1, indicating that the internal regional innovations of each block had significant connectedness. This image matrix clearly shows the delivery mechanism of urban innovation of Guangdong. Block I transmits the momentum of urban innovation through Blocks II and IV. Blocks III and IV transfer the dynamics of urban innovation to each other. Block I does not directly influence the third plate, but transmits to Block III through Block IV. This transmission mechanism is characterized by a clear “gradient” spillover. Cities of Block I, the engine of urban innovation, are all located in a developed area in the Pearl River Delta or along the coast, having a spillover effect and a driving effect on lagging areas.

**Table 14:** Image matrix of blocks.

	Block I	Block II	Block III	Block IV
Block I	1	1	0	1
Block II	0	1	0	0
Block III	0	0	1	1
Block IV	0	0	1	1

## Conclusions and recommendations

### Conclusion

In this paper, the spatial pattern and association of urban innovation of Guangdong is measured from 2008–2017. Moran’s I and SNA are used to deconstruct the spatial correlation characteristics of urban innovation in Guangdong in a new way. In the following, the major conclusions are summarized:

1. The Moran’s I of Guangdong’s urban innovation from 2008 to 2017 not only passed the 10% significance test, but also increased year by year. This indicates that the innovation linkage between the cities of Guangdong has gradually increased, and spatial agglomeration has followed an enhancing trend. The network density of urban innovation in Guangdong increased from 0.2405 in 2008 to 0.2857 in 2017, and the correlation increased from 0.596 in 2008 to 0.701 in 2017. This shows that there is a general spatial spillover relationship and a clustering or diffusion effect of urban innovation in Guangdong, which exhibits spatial correlation.
2. In 2017, the overall density of the spatial correlation network of urban innovation in Guangdong was low at 0.267. This indicates that the closeness of the spatial correlation of innovation among individual cities is low. The correlation degree of the spatial correlation network of urban innovation is 1, reaching its maximum. The linkage effect of the spatial correlation network of urban innovation in Guangdong is marginally significant.
3. The spatially correlation network of urban innovation in Guangdong contains considerable unevenness, and the unevenness of the beneficial effect outweighs the unevenness of the spillover effect. Each city has a different status and role in the network. Centrality analysis showed that Foshan, Meizhou, Zhongshan, and Maoming, which are most associated with other cities, rank high in correlation relationships and play the role of “bridges” and “transmitters”. The Pearl River Delta has the highest spatial spillover effect.
4. Guangdong’s urban innovation spatial correlations show a gradient. Block I is the engine of urban innovation and transmits the energy of urban innovation through Blocks II and IV. Block IV acts as a bridge. Block IV transmits the energy of urban innovation to Block III. At the same time, Block III transmits the energy of urban innovation to Block IV. Block I, as the engine, includes the developed areas of the Pearl River Delta or the coast, which exerts both a spillover effect and a driving effect on backward areas.

### Recommendations

Firstly, it is necessary for the government to consider urban spatial correlation as an important decision-making variable for coordinated regional development. Administrative barriers should be removed, institutional safeguards implemented, barriers to innovation broken down, and the closeness of linkages between cities increased. Moreover, more spatial spillover ‘pipelines’ should be created and sustainable growth in innovation capacity should be achieved.

Secondly, it is important to select targeted urban development policies to address the different statuses and roles of cities in spatial correlation and the different functions of innovation segments. Targeted and precise regulation should be carried out to enhance the spatial synergy of urban innovation. The government should not only focus on cities with strong control over resources and two-way spillover innovation plate to further stimulate the “powerhouses” of spatial spillover effects, but also “warm up” cities and “intermediaries” that play an important role in urban innovation. Regions that play an important “intermediary” role should also be “warmed up” and become members of the agent plate in urban innovation to further enhance the transmission function of these regions. Also, Guangdong should continue to provide a quality innovation environment and create a good reception platform for “pipeline” cities and beneficiary cities.

Third, Guangdong should strengthen the degree of urban innovation correlation, taking full advantage of the spatial correlation generated by geographically adjacent areas and similar levels of innovation. The development of overall regional innovation capabilities should be coordinated and the differences in innovation conditions between cities should be reduced. Amplifying the spatial spillover effects of urban innovation is of great significance.

Fourth, Guangdong should strengthen cooperation and exchange between urban innovation agents. Extensive exchanges among enterprises, talents, research institutions, universities, and social groups enable the free circulation of innovation factors within each city. Consequently, a trend towards higher innovation intensity is created to lead the development of cities with lower innovation intensity towards realizing synergistic development of innovation between regions.
